# Programmatic adaptations to acute malnutrition screening and treatment during the COVID‐19 pandemic

**DOI:** 10.1111/mcn.13406

**Published:** 2022-08-05

**Authors:** Maria Wrabel, Ronald Stokes‐Walters, Sarah King, Grace Funnell, Heather Stobaugh

**Affiliations:** ^1^ Action Against Hunger USA Washington, DC USA; ^2^ UNICEF New York New York USA; ^3^ Friedman School of Nutrition Science and Policy Tufts University Boston Massachusetts USA

**Keywords:** assessment of nutritional status, community‐based, community‐based management of acute malnutrition (CMAM), COVID‐19, Family MUAC, infant and child nutrition, low‐income countries, malnutrition, programme components, simplified approaches, wasting

## Abstract

The COVID‐19 pandemic presented numerous challenges to acute malnutrition screening and treatment. To enable continued case identification and service delivery while minimising transmission risks, many organisations and governments implemented adaptations to community‐based management of acute malnutrition (CMAM) programmes for children under 5. These included: Family mid‐upper arm circumference (MUAC); modified admission and discharge criteria; modified dosage of therapeutic foods; and reduced frequency of follow‐up visits. This paper presents qualitative findings from a larger mixed methods study to document practitioners' operational experiences and lessons learned from these adaptations. Findings reflect insights from 37 interviews representing 15 organisations in 17 countries, conducted between July 2020 and January 2021. Overall, interviewees indicated that adaptations were mostly well‐accepted by staff, caregivers and communities. Family MUAC filled screening gaps linked to COVID‐19 disruptions; however, challenges included long‐term accuracy of caregiver measurements; implementing an intervention that could increase demand for inconsistent services; and limited guidance to monitor programme quality and impact. Modified admission and discharge criteria and modified dosage streamlined logistics and implementation with positive impacts on staff workload and caregiver understanding of the programme. Reduced frequency of visits enabled social distancing by minimising crowding at facilities and lessened caregivers' need to travel. Concerns remained about how adaptations impacted children's identification for and progress through treatment and programme outcomes. Most respondents anticipated reverting to standard protocols once transmission risks were mitigated. Further evidence, including multi‐year programmatic data analysis and rigorous research, is needed in diverse contexts to understand adaptations' impacts, including how to ensure equity and mitigate unintended consequences.

## Key messages

- COVID‐19 CMAM programme adaptations enabled service continuity despite pandemic‐related challenges. Further evidence is needed on long‐term impacts.
- Family MUAC was well‐accepted and addressed screening gaps from COVID‐19 disruptions. Challenges included sustaining caregiver measurement accuracy; handling inaccurate self‐referrals to encourage health‐seeking behaviours; and limited programme design and monitoring guidance and tools.
- Modified admission criteria and therapeutic food dosage reduced contact between staff and children and streamlined logistics and implementation. Concerns remained about effects on programme admissions and outcomes.
- Reduced frequency of follow‐up visits successfully reduced facility crowding and need for caregiver travel. However, infrequent monitoring of childrenmay miss deterioration.

## 1 INTRODUCTION

Acute malnutrition remains a critical global challenge: an estimated 45.4 million children under 5 suffered from acute malnutrition in 2020 (United Nations Children's Fund World Health Organisation WHO & World Bank Group, 2021). Children with severe or moderate acute malnutrition (SAM or MAM) are at significantly increased risk of death and morbidity compared to nonmalnourished children (Black et al., 2008), with long‐term impacts on physical and cognitive development. The COVID‐19 pandemic is projected to exacerbate this challenge: estimates indicate that up to 9.3 million additional children may suffer from acute malnutrition due to economic impacts, increased food insecurity and interrupted health service provision (Headey et al., 2020; Osendarp et al., 2021).

Community‐based management of acute malnutrition (CMAM) is the standard of care for SAM and MAM treatment. This approach consists of four components: community outreach and screening; outpatient MAM treatment through targeted supplementary feeding programmes; outpatient SAM treatment through outpatient therapeutic feeding programmes; and inpatient treatment of SAM with medical complications in hospitals or stabilisation centres. CMAM programme referrals are achieved through clinic‐based screening and community‐based screening, either through mass campaigns or household visits by trained community health workers/volunteers (CHWs/Vs) (ALIMA, 2016).

However, standard CMAM protocols may pose an increased risk of COVID‐19 transmission for health workers, caregivers and patients through prolonged proximity during screening processes and frequent trips to crowded health facilities. Furthermore, pandemic‐related movement restrictions and social distancing requirements made many standard community‐based screening and referral methods impossible.

To enable continued service provision while reducing transmission risk, the United Nations Children Fund (UNICEF), Global Nutrition Cluster (GNC) and the Global Technical Assistance Mechanism for Nutrition (GTAM) released a series of guidance briefs regarding potential adaptations to outpatient acute malnutrition treatment protocols in March 2020 (UNICEF, Global Nutrition Cluster, & Global Technical Assistance Mechanism for Nutrition, 2020); later, UNICEF and the World Health Organisation (WHO) released further implementation guidance (UNICEF & WHO, 2020). In addition to infection prevention and control (IPC) measures and use of personal protective equipment (PPE), programme protocol adaptations were recommended, including changes to screening processes; programme admission and discharge criteria; dosage of therapeutic foods for admitted children; and the frequency with which children return to health facilities for follow‐up visits throughout care, among others.

Many adaptations, sometimes referred to as ‘simplified approaches’ to CMAM, were previously implemented in research settings or piloted during emergencies to improve access to and quality of acute malnutrition treatment (Action Against Hunger USA, 2021). However, questions remain regarding feasibility, operational implications and effectiveness in varied contexts. The mass rollout of these adaptations as the COVID‐19 pandemic escalated in early 2020 presented a unique opportunity to examine them at a scale larger than ever before. Therefore, Action Against Hunger USA, in partnership with the United States Agency for International Development (USAID), led a study to document lessons learned from CMAM protocol adaptations during the COVID‐19 pandemic.

## 2 METHODS

This paper presents the qualitative findings from a larger mixed methods study conducted to identify, document and analyse protocol adaptations during the COVID‐19 pandemic (Action Against Hunger USA, 2022). The target population for this study included global, national and subnational humanitarian aid practitioners from multilateral international organisations and nongovernmental organisations (NGOs). The research team was comprised of four Action Against Hunger staff, including one researcher with experience in CMAM implementation and qualitative analysis, one trained in qualitative analysis and two with extensive experience in acute malnutrition research.

### 2.1 Sampling strategy and data collection

Interviewees were identified from respondents to a high‐level survey, disseminated globally through practitioner networks, nutrition coordination mechanisms and NGOs, targeting stakeholders with specialised technical or operational knowledge of CMAM programmes and accompanying adaptations. The screening survey included simple questions about which adaptations were being implemented where and by whom; when adaptations started; and anticipated duration. Survey responses were collected from July 2020 to January 2021; summary statistics were published on a rolling basis on the State of Acute Malnutrition website to inform practitioners and policymakers of adaptation prevalence (No Wasted Lives, 2021). Respondents who consented to be contacted within the survey were invited to participate in interviews. Additional interview participants were identified through a combination of purposive sampling (based on geographic location or adaptations knowledge) and snowball sampling. Interviews were conducted between July 2020 and January 2021.

### 2.2 Interviews

A semistructured interview guide was developed based on research questions identified as key evidence gaps through policymaker and practitioner consultations on CMAM programme adaptation implementation during the pandemic and simplified approaches more generally. These questions addressed the following themes: decision‐making actors, processes and factors; staff and caregiver training; operational considerations (e.g., staffing, logistics, costs); and strengths, challenges and lessons learned, including perceived impacts on programme performance (e.g., admissions, programme outcomes). Each interview, conducted in English, verified survey responses and discussed the points above for each adaptation applied in the interviewee's area of work. Two research team members attended each interview, serving as an interviewer and a notetaker, respectively. The interview team discussed key findings and themes for further investigation after each interview and included these in summary memos.

### 2.3 Data analysis

Recorded interviews were transcribed and qualitative data were analysed using thematic analysis, a flexible qualitative analysis method that identifies patterns across interviews corresponding to deductively and inductively identified themes (Braun & Clarke, 2008). The four research team members developed a deductive codebook derived from the research questions and an initial literature review on simplified approaches, focusing on operational components of programme implementation (Action Against Hunger USA, 2021). Inductive codes for new themes or notable subthemes were also added as necessary during analysis. Two team members with training and experience in qualitative research methods conducted data analysis using Dedoose qualitative analysis software, triangulating findings with interview summary memos. The first four transcripts were double coded by two reviewers independently to verify coder agreement. Subsequently, one out of five transcripts was randomly selected for double coding.

After coding all interviews, code reports were generated to show tagged quotations for each parent code in the codebook, organised by the corresponding adaptation. These quotes were reviewed and used to identify key findings, defined either as (1) observations identified consistently across multiple geographies; or (2) context‐specific factors that significantly impacted implementation. Coders developed iterative adaptation‐specific analytical matrices, which identified takeaways for each theme, triangulated findings across interviews and contexts, and identified irregularities, contradictions and points for further investigation.

### 2.4 Ethical approval and considerations

Ethical approval was obtained from Solutions IRB, a private accredited Institutional Review Board (Reference #2020/06/18). All participants provided written informed consent to participate and be recorded before any data collection. Participants were informed of the study objectives and intended usage of findings, as well as the principles of voluntary participation and right to withdraw. To ensure anonymity, each interview was assigned a unique reference number and identifiable information removed before analysis.

## 3 RESULTS

In total, the research team conducted 43 interviews; six interviews were excluded from analysis: two due to a misalignment with study objectives, and four due to corrupted recording files. Findings therefore reflect analysis of 37 interviews representing 15 organisations in 17 countries across East Africa, West and Central Africa, Southeast Asia, South Asia and the Middle East (Table 1). Of the individuals interviewed, one represented their organisation or project at a global level; 23 held national‐level positions (e.g., head of department, chief of party, etc.); and 13 held subnational positions (e.g., programme manager, nutrition associate, etc.).

**Table 1:** Organisations and countries represented in analysed interviews

Organisations represented	Countries represented
Accion Contra el Hambre^a^	Bangladesh
Action Against Hunger India	Democratic Republic of Congo (DRC)^a^
Action Against Hunger USA	Ethiopia
Action Contre la Faim	India
Adventist Development and Relief Agency (ADRA)	Jordan
Alliance for International Medical Action (ALIMA)*	Kenya
Catholic Relief Services	Malawi
Concern Worldwide	Myanmar
International Medical Corps	Nepal
National Drought Management Authority (NDMA)	Nigeria
Kenya Red Cross	Pakistan
Save the Children	Philippines
United Nations High Commissioner for Refugees (UNHCR)	Somalia
United Nations Children's Fund (UNICEF)	South Sudan
World Vision	Tanzania
	Uganda
	Yemen

^a^ Denotes organisation interviewed regarding operational research on simplified approaches.

Decisions about implementing CMAM adaptations were largely informed by UNICEF's global‐level guidance. National Ministries of Health typically led country‐specific conversations, with input from Nutrition Clusters, UNICEF, the World Food Programme (WFP), technical working groups, and international and local NGOs. In refugee settlements or camps, the United Nations High Commissioner for Refugees (UNHCR) was also engaged. Decision‐making was typically a multi‐step process with several rounds of consultation and revision, with policies cascaded from national level to facilities and communities.

The following sections discuss findings related to each of the four most discussed adaptations: Family mid‐upper arm circumference (MUAC); modified admission and discharge criteria; modified dosage of therapeutic foods; and reduced frequency of follow‐up visits. For each adaptation, the analysis focuses on decision‐making factors, operational considerations, and strengths, challenges and opportunities.

### 3.1 Family MUAC

The Family MUAC approach equips caregivers with the skills to measure their children's MUAC, a common indicator for detecting acute malnutrition, using simple colour‐coded MUAC tapes. Through an initial and refresher trainings, Family MUAC trains and motivates caregivers to monitor their child's nutritional status regularly, enabling earlier detection of and referral to treatment for acute malnutrition (ALIMA, 2016).

Family MUAC was implemented in numerous contexts before COVID‐19 (The State of Acute Malnutrition, 2019); however, the approach was rapidly scaled up during the pandemic when regular nutrition screening and referral programmes were suspended. There was usually widespread stakeholder consensus to implement the approach. Family MUAC was cited as an acceptable strategy with suspended community screenings by interviewees in Uganda, India, Yemen, Tanzania, Somalia and Kenya. Elsewhere, such as in Bangladesh, Family MUAC complemented ongoing mass screening services. Key findings are summarised in Table 2.

**Table 2:** Key findings: Family MUAC

Preparing to implement	Operational considerations	Strengths, challenges and opportunities
**Decision‐making factors**: Reducing physical contact between caregivers, children, and healthcare workers Replacing suspended mass screenings or complementing limited community‐level screening Responding to anticipated or perceived increases in acute malnutrition Complying with issued global guidance Identifying alternative sources of data for community surveillance systems	**Training**: Implementation of quality trainings and close collaboration with communities critical to build long‐lasting support Simple and engaging training materials targeted to low‐literate audiences highly recommended Common caregiver training modalities: care groups, one‐on‐one training at household level, small groups Some higher training costs for Family MUAC compared to traditional CHW‐led approaches with adherence to COVID‐19 protocols to limit gathering sizes; virtual trainings reduced some costs **Staffing and workload**: Shifted CHW responsibilities from screening to caregiver follow‐up, refresher trainings and supervision, and measurement validation Limited incentives available for CHWs to account for increased responsibilities **Logistics and cost implications** Substantial delays in MUAC tape procurement due to supply chain issues and unclear lines of procurement responsibility Limited funds initially available for rapid scale‐up of trainings; later built into programme budgets	**Strengths**: Highly valued as an alternative or supplementary screening strategy High community acceptance due to simplicity and perceived value Strong programme staff acceptance due to the approach's focus on knowledge transfer, capacity building and increased community engagement **Challenges**: Absence of guidance and materials on designing and implementing a Family MUAC programme early in the pandemic Some caregiver reluctance due to low confidence about their own capacity and expectations for CHWs to take the measurements Staff concerns around accuracy of caregiver measurements Limited standardised reporting and/or monitoring and evaluation systems, including tools to capture referral sources at facilities, hindering ability to demonstrate Family MUAC's impact on programme admissions and outcomes Lack of treatment for MAM cases identified through Family MUAC in contexts without ongoing SFP programming COVID‐specific challenges: movement restrictions and lockdowns; PPE shortages **Opportunities**: Strong perceived likelihood of Family MUAC approach continuing beyond the pandemic

Abbreviations: CHW, community health worker; MUAC, mid‐upper arm circumference; PPE, personal protective equipment.

#### 3.1.1 Operational considerations

Interviewees emphasised the importance of engaging caregivers as an essential component of a successful Family MUAC intervention, requiring high‐quality trainings and building long‐lasting community buy‐in. Interviewees noted that training materials and messages should be simple and engaging, particularly for audiences with limited or no literacy. Additionally, Family MUAC acceptance was more successful when trainers shared similar linguistic, cultural and social backgrounds as the trainees.

“We have a presentation which we made really simple for the village level health workers, so they can easily roll that out to the parents. And so we made the language pretty easy for them to understand … For the parents, we need to emphasize that they need to look at the color coding …. But it's important that we catch these children early and even before. If we can prevent, that's much better.” (NGO Practitioner, Somalia)

“There will be external people, but it will be easier for us to penetrate the communities using [local] volunteers because they know better the languages and the culture. They know how to explain things, which maybe we wouldn't be able to explain.” (NGO Practitioner, Uganda)

Caregiver training modalities differed across contexts, including delivery through existing group platforms (e.g., Care Groups) (Perry et al., 2015; SPRING Group, 2015), individually at household level, directly at health facilities, or through community‐based small group training sessions. Because standard Family MUAC training requires proximity between trainers and trainees, trainers identified creative solutions to observe social distancing during trainings: using ripe fruit to demonstrate bilateral pitting oedema assessment (a clinical sign of acute malnutrition); practicing MUAC measurements on tissue paper rolls and dolls; and demonstration videos. Interviewees noted higher initial training costs compared to traditional CHW‐led screening approaches, due to increased trainings to comply with COVID‐19 IPC guidance limiting gathering sizes. However, using virtual trainings reduced costs elsewhere.

Interviewees generally agreed that Family MUAC reduced CHW workload. Still, CHWs assumed other responsibilities: caregiver follow‐up, refresher trainings, supervisory visits, and validating caregiver measurements at the community level. Given this shift, it was critical to clearly define caregivers' and CHWs' roles and responsibilities. Another concern was the challenge in ensuring that CHWs received appropriate incentives or compensation for these additional responsibilities, a key barrier to scale up.

Family MUAC rollout also faced pandemic‐related logistical challenges, particularly the inability to acquire sufficient MUAC tapes to meet increased training needs early in the pandemic. Extended procurement and shipment times due to lockdowns, travel restrictions and demand compounded these challenges. Furthermore, overlapping and occasionally unclear lines of procurement responsibility added to challenges.

“We were having a big challenge to get the MUAC tapes in the country … due to [sic] covid … so we were really struggling to get the MUAC tapes to implement the [sic] training because we have a target of 24,000 beneficiaries …” (NGO Practitioner, Yemen)

Finally, interviewees also noted several budgetary implications, including additional funds needed to support Family MUAC trainings (travel, supplies, etc.) and increased MUAC tape stocks, which were not included in pre‐COVID budgets. Ultimately, the pandemic accelerated Family MUAC implementation to the extent that some interviewees noted that it was a standard component of proposal development, with associated costs routinely included in project budgets.

#### 3.1.2 Strengths, challenges and opportunities

Some interviewees cited initial resistance to Family MUAC, including caregivers' lack of confidence to correctly measure their child, and community expectations that CHWs were solely responsible for taking these measurements. However, Family MUAC was quickly accepted by community members due to its simplicity. One interviewee noted that despite a long‐standing wariness of all health interventions, communities saw Family MUAC's value and adopted it enthusiastically.

“Initially, most of the mothers would say, ‘This is not our work. This is the work of frontline workers to measure our child. Why [would] we do this?’ This took some time to explain [to] them that ‘this is your child. And [frontline workers] will support you. You can identify your child early and your child [will] not fall in the status of malnutrition’.” (NGO Practitioner, India)

Most interviewees indicated that programme staff also largely accepted Family MUAC, appreciating its focus on knowledge transfer, capacity‐building and increased community engagement in acute malnutrition management.

“My team and I saw how the health workers became more interested in doing management of acute malnutrition …. [and] when we involve the barangay [community] health workers … they were more active in terms of reaching out to these parents and families with children who are wasted.” (NGO practitioner, Philippines)

Despite overall acceptance, interviewees flagged some staff concerns, the most frequent being caregiver measurement accuracy. Though programmatic data were unavailable at the time of interviews, interviewees anecdotally estimated anywhere between 30% and 70% of caregiver referrals were accurate, with caregivers reportedly tending to apply the MUAC tape too tightly. This highlights the importance of continued refresher trainings and monitoring to maintain caregiver skill and accuracy. Interviewees also cautioned that inaccurate measurements could have longer‐term negative effects, whereby caregivers who incorrectly self‐refer their children and are turned away at facilities may be reluctant to return.

“We keep monitoring to keep refreshing the information, because we agree that taking MUAC is very easy to learn, but also easy to fail.” (NGO Practitioner, Uganda)

“What we want to avoid as much as possible is caregivers referring false positives, … because this would result in a lot of disgruntlement by the caregivers, if they see that their children are not being admitted or not being treated.” (NGO Practitioner, Malawi)

A final challenge multiple interviewees noted was the relative absence of guidance and materials on designing a comprehensive Family MUAC programme, leaving most organisations and agencies to develop ad hoc programmes. Available guidance was largely limited to MUAC measurement training methods, rather than for programme design, implementation and monitoring. Furthermore, monitoring and evaluation systems lagged in both maturity and spread, including a lack of standard reporting tools and infrastructure, limited financial resources, and insufficient data quality monitors. These challenges were critical barriers to capturing referrals and measurement accuracy and demonstrating the impact of Family MUAC on admissions and programmatic outcomes. Interviewees recommended identifying standard process indicators, outcome indicators, and qualitative data capturing caregiver feedback, and adding these indicators to standard reporting forms to encourage routine data collection.

Overall, most interviewees noted that while COVID‐19 initially reduced programme admissions, they anecdotally observed an increase once Family MUAC was implemented, though other factors may have influenced this change. However, many cases were identified in areas with limited treatment options, particularly for children identified with MAM in contexts without SFP treatment, presenting an ethical challenge. Furthermore, other factors, such as distance to facilities, remained barriers to accessing treatment.

“We did Family MUAC, but we did not have an increase in number of children being admitted to the CMAM program. The camps are really big and the distance for walking is really long.” (NGO Practitioner, Jordan)

Despite these challenges, interviewees were generally positive about the approach and hopeful for its continued improvement and potential impact. Most interviewees expressed confidence that Family MUAC would continue to be implemented beyond the COVID pandemic. Multiple interviewees noted that the approach was widely discussed within coordination mechanisms, and saw resources shifted towards Family MUAC. Even in contexts with limited government buy‐in, interviewees expressed confidence that the value of Family MUAC would eventually earn government approval.

“I think Family MUAC is the way to go. What I've seen is that caregivers want to be empowered and they have the best interest for their children.” (NGO Practitioner, Kenya)

### 3.2 Modified admission and discharge criteria

In standard CMAM programme protocols, three criteria are typically used to determine admission: MUAC; weight‐for‐height Z‐score (WHZ), calculated using a child's weight and height measurements; and bilateral pitting oedema. During the pandemic, two adaptations to these criteria were made. Several programmes paused the use of WHZ as staff suspended weight and height measurements to reduce contact with children and mitigate viral transmission (reported as implemented by 16 interviewees in Bangladesh, Ethiopia, Somalia [briefly], South Sudan, Tanzania, Uganda). A second adaptation increased the MUAC threshold for admission eligibility to capture children otherwise eligible by the now‐suspended WHZ (reported as implemented by four interviewees in Bangladesh and Uganda). Several interviewees commented that suspending WHZ took place alongside implementation of other adaptations, such as Family MUAC, and later intensified house‐to‐house screening once movement restrictions abated and PPE was procured (Bangladesh, South Sudan, Tanzania, Uganda).

In addition to reducing contact between staff and patients, interviewees commented that suspending WHZ aimed to streamline and reduce caregivers' and children's time at the sites, minimising crowding and maximising patient flow. Furthermore, using only MUAC and oedema as admission criteria aligned with existing evidence pointing to low MUAC as a better predictor of mortality in undernourished children (Briend et al., 2016; Rasmussen et al., 2012). However, some interviewees reported concerns that eliminating WHZ would exclude children with low WHZ who might benefit from treatment, since MUAC and WHZ do not always identify the same children (Grellety et al., 2015; Laillou et al., 2014). In Somalia, a significant drop in admissions motivated a reversion to standard admission criteria shortly after the adaptation was implemented.

To address the concerns of potentially excluding children who might benefit from treatment, some interviewees reported high‐level discussions about expanding the MUAC admission threshold. Decision makers analysed existing programme and screening data to identify MUAC thresholds that would also capture children otherwise identified by WHZ. This determination was particularly challenging in contexts with high discordance between acute malnutrition identified by WHZ and MUAC, meaning that not all children otherwise eligible by WHZ would be included. Another decision‐making factor was whether sufficient resources were available to accommodate increased caseloads, as higher MUAC thresholds can expand caseloads beyond just children who would have been captured via WHZ (Guesdon et al., 2021). Table 3 summarises key findings for both modifications.

**Table 3:** Key findings: Modified admission and discharge criteria

Preparing to implement	Operational considerations	Strengths, challenges and opportunities
**Decision‐making factors: Suspending WHZ** Reducing contact between staff and patients Streamlining caregivers' and children's time at health facilities Concerns: excluding nutritionally vulnerable children **Decision‐making factors: Expanding admission criteria** Capturing children otherwise identified by the suspended WHZ criterion Concerns: increasing caseloads beyond capacity of existing resources	**Staffing and workload**: Suspending WHZ eased staff's workload, mitigated increased demands from other adaptations and IPC measures **Logistics and cost implications**: Suspending WHZ reduced demand for expensive weighing scales and height boards; increased demand for less expensive MUAC tapes Expanded admission criteria increased caseloads and consumption of therapeutic foods	**Strengths**: Staff relieved by reduced physical contact during pandemic Expanding admission criteria enabled continued enrolment of some nutritionally vulnerable children despite WHZ suspension **Challenges**: Staff concerned that suspending WHZ measurements without also increasing MUAC thresholds excluded or misclassified some children Expanding admission criteria unsustainably increased caseloads in some contexts **Opportunities**: Improving community outreach and household visits alongside changes to admission criteria anecdotally increased caregiver support and adherence

Abbreviations: IPC, infection prevention and control; MUAC, mid‐upper arm circumference; WHZ, weight‐for‐height Z‐score.

#### 3.2.1 Operational considerations

Interviewees generally reported that suspending WHZ eased staff's workload, with positive feedback on the simpler methods, ease of training and straightforward explanation of admission criteria to caregivers. The reduced workload also reportedly mitigated increased demands from other adaptations and additional reporting requirements during the pandemic.

“[Suspending WHZ] has been like a reduced workload. The [staff] response has been positive, because when we are conducting MUAC it is a bit easier than doing again the weight‐for‐height and then taking Z‐scores, because MUAC is more simple.” (NGO Practitioner, Tanzania)

Across different contexts, expanding the MUAC threshold for admission increased caseloads, with corresponding logistical challenges. In one context, decision‐makers indicated that, despite the increased demand on resources, they preferred to admit ‘too many children’ rather than risk excluding children. Elsewhere, an interviewee estimated that raising the MUAC threshold for admission could increase the consumption of therapeutic foods up to 30%. This would require increased supplies, resources and budgets, which ultimately led decision makers to maintain the standard MUAC threshold.

“Increasing the cutoffs again helped us enroll more children. And even if there were some children who may not be SAM or MAM with this increased cutoff, at least we are not doing any harm to them.” (NGO Practitioner, Bangladesh)

#### 3.2.2 Strengths, challenges and opportunities

Despite a few challenges in rollout, interviewees reported that staff were relieved by reduced physical contact with patients. They also indicated that caregivers accepted the suspension of WHZ because a MUAC measurement is a less distressing experience for the child than collecting weight and height measurements.

“[Caregivers] are a bit happy because they don't have to do the weight‐for‐height because their babies used to cry a lot during that.” (NGO Practitioner, Uganda)

Anecdotal reports indicate that suspending WHZ without a simultaneous increase in MUAC admission thresholds was associated with decreased admissions, possibly due to impacts on eligibility in contexts with high acute malnutrition by WHZ. However, interviewees also proposed that other factors could drive reduced admissions during the pandemic, including fear of visiting facilities, suspended mass screenings and reduced house‐to‐house screenings and referrals. Two interviewees were concerned that shifting to MUAC and oedema only would identify MAM in some children who would otherwise have been categorised as having SAM by WHZ; therefore, such children may not receive appropriate treatment.

“A lower level of admission trend doesn't mean that malnutrition has gone out from South Sudan… The malnutrition is still there. And it has been growing worse because of some conditions under this COVID‐19 pandemic.” (NGO Practitioner, South Sudan)

Concurrent implementation of Family MUAC and expanded MUAC thresholds also presented a discordant challenge, as the standard colour‐coded MUAC tapes that caregivers are trained to use do not show the increased threshold. Interviewees were concerned that relying on self‐identification using standard tapes despite an expanded admission threshold could exclude eligible vulnerable children. Most interviewees implementing both adaptations reported continuing to train caregivers on standard thresholds for lack of a better option, while one commented that they marked the new threshold on each MUAC tape by hand.

Overall, interviewees commented that expanding MUAC thresholds enabled continued enrolment of nutritionally vulnerable children with suspended WHZ. Despite increased caseloads, interviewees reported limited perceived impact on programme performance. One interviewee noted anecdotally that the expanded discharge threshold increased length of stay (LOS) during the first few months of implementation, as children required a longer treatment period to reach an increased discharge threshold. Their organisation addressed this by improving outreach, monitoring and household visits, using PPE, to ensure that caregivers had adequate support and were adhering to treatment protocols.

### 3.3 Modified dosage

Under standard protocols, ready‐to‐use therapeutic food (RUTF) dosage for SAM children is calculated from a child's weight; however, during the pandemic, many organisations suspended weight measurements to reduce contact. Therefore, a common COVID‐19 CMAM programme adaptation was to modify RUTF dosage. Overall, eight interviewees from South Sudan, Bangladesh and Tanzania reported implementing modified dosage protocols during the pandemic; two interviewees reported on non‐COVID research projects on modified dosage in Democratic Republic of Congo and Kenya. Modifications included: a universal dosage for all SAM cases based on previous research findings (Bailey et al., 2020); weight‐based calculations for enroled children using their last recorded weight and a universal dosage for new cases; and case‐specific calculations (e.g., dosage based on age, appetite, etc.). Table 4 summarises key findings.

**Table 4:** Key findings: Modified dosage of therapeutic foods

Preparing to implement	Operational considerations	Strengths, challenges and opportunities
**Decision‐making factors**: Substituting for weight‐based dosage calculations in the context of suspended weight measurements Streamlining dosage calculations Reducing time caregivers spent at sites, enforcing capacity limits and social distancing measures	**Staffing and workload**: Simplified ration size calculation and enabled preparation in advance of caregiver visits **Logistics and cost implications**: Simplified stock management and forecasting	**Strengths**: Well received by staff to enable service provision despite suspended weight measurements **Challenges**: Concerns about negative impacts of a modified dosage on recovery General caregiver perceptions of insufficient rations

#### 3.3.1 Operational considerations

Respondents reported that modified dosage protocols were quicker and easier, successfully reducing children and caregivers' time at sites. For example, using a universal, standardised dosage (e.g., two sachets per child per day) enabled staff to prepare rations in advance. This further simplified staff workload and enabled easier training of new staff. Interviewees indicated that modified dosage simplified stock management, both in maintaining stocks longer due to the reduced rations distributed and simplifying forecasting calculations for procurement.

“Previously [staff] used to check the lookup table for RUTF because it is different from child to child. But currently they don't need to check, so they feel like it's really very quick. We know how many we are giving for each beneficiary. So they just count and give.” (NGO Practitioner, South Sudan)

#### 3.3.2 Strengths, opportunities and challenges

Overall, interviewees reported that staff appreciated the adaptation as it enabled continued service provision despite suspended weight measurements, streamlined stock management and improved patient flow. One interviewee in South Sudan commented that the adaptation enabled treatment of more children since they used less product.

Notwithstanding prospective improvements to supply management and distribution, interviewees were concerned about potential negative impacts on children's progress and programme performance. Interviewees noted that both staff and caregivers were consistently concerned that a reduced dosage would be insufficient for recovery. However, interviewees also proposed other exacerbating factors, such as simultaneous adaptations and increased intrahousehold sharing of the reduced supply due to pandemic‐related food insecurity.

“…A [caregiver] may give [RUTF] to her [non‐malnourished] children at household level. And that might be a contributing factor for non‐response rate.” (NGO Practitioner, South Sudan)

Despite initial frustration in receiving smaller rations, caregivers gradually accepted the adaptation with thorough explanation. However, caregiver dissatisfaction with ration size may be unrelated to the adaptation itself, instead reflecting their desire to acquire more food given severe food insecurity: one interviewee representing a modified dosage study in Kenya commented that caregivers receiving either the regular or modified dosage were dissatisfied with the amount. Due to these concerns and the intensive high‐level discussions necessary to modify dosage protocols in national‐ and global‐level policies, most interviewees anticipated eventually returning to standard weight‐based dosage.

### 3.4 Reduced frequency of follow‐up visits

In typical CMAM programmes, children with SAM return weekly to facilities for monitoring and food distribution, while children with MAM return biweekly. During the pandemic, the frequency of these return visits was reduced to control crowding at health facilities and enable social distancing. Many organisations therefore shifted SAM visits from weekly to biweekly and MAM visits from biweekly to monthly, resulting in fewer overall visits, longer periods between visits, and thus larger ration sizes distributed at each visit. The research team interviewed 23 staff from 12 countries (Bangladesh, Ethiopia, Jordan, Malawi, Myanmar, Nepal, Pakistan, Somalia, South Sudan, Tanzania, Uganda and Yemen) who implemented this adaptation during COVID‐19 (see Table 5 for a summary of key findings).

**Table 5:** Key findings: Reduced frequency of follow‐up visits

Preparing to implement	Operational considerations	Strengths, challenges and opportunities
**Decision‐making factors**: Enabling social distancing at health facilities by controlling crowd size Accommodating caregivers' fears of contracting COVID‐19 during frequent health facility visits Concern: potential for rapid deterioration of SAM children without frequent follow‐ups; option to determine frequency of visits on an individual basis	**Staffing and workload**: Variable impacts on staff workloads: reduced facility‐level workloads; increased community‐level tasks and follow‐up **Logistics and cost implications**: Increasing ration size quickly drained supplies during initial rollout Limited perceived long‐term changes to stock management	**Strengths**: Reduced crowding at facilities Alleviated burden on caregivers to travel long distances, especially during lockdowns **Challenges**: Concerns about caregivers' capacity to manage increased rations Reports of increased sale and intrahousehold ration sharing, especially among food‐insecure households Anecdotal observations of increased defaulter rates and deterioration from less frequent monitoring

#### 3.4.1 Operational considerations

Reducing frequency of follow‐up visits for children during treatment impacted staff workloads variably. Reduced crowds sometimes alleviated facility‐based workloads, allowing staff to dedicate more time to community engagement and household visits, contingent on PPE availability. Others commented that this instead elevated staff workloads due to increased absentee or defaulter tracing and door‐to‐door dissemination of messages and appointment information. Either impact on workloads could influence staffing needs and budgets: increased community‐based work would require additional transportation, while lengthened working days could warrant an increase in staffing or remuneration.

Supply chain management and logistics impacts were primarily felt during rollout. In some cases, increasing ration sizes at each visit quickly depleted available supplies, as forecasts and procurement requests were previously based on weekly or biweekly calculations. One interviewee initially reported needing increased staff for supply chain management, while another reported more streamlined stock management from procuring more supplies at once. Ultimately, the amount of product distributed remained the same but was distributed differently. Flexible supply chains and contingency plans were cited as key enabling factors to ease the transition to less frequent but larger distributions.

“Due to this change, our supplies were consumed quicker and even though we placed international order of more supplies well in advance … international suppliers took longer than usual and therefore our supplies went into a delay.” (NGO Practitioner, Pakistan)

#### 3.4.2 Strengths, opportunities and challenges

Overall, this adaptation achieved its primary aim of reducing facility crowding, thereby minimising potential exposure to COVID‐19. One interviewee in Malawi called this a ‘blessing in disguise’, observing that the approach could be particularly useful in contexts with a low prevalence of acute malnutrition and extended distances to facilities. Interviewees also indicated that communities and caregivers accepted this adaptation well, as it alleviated the challenges associated with competing household responsibilities, transportation costs and traversing difficult terrain.

At each visit, caregivers are provided with a ration of therapeutic or supplementary food to last until the next visit. Reducing visit frequency therefore increased ration sizes, which caregivers reportedly struggled to manage in some contexts. This included storing and protecting the ration, particularly in a refugee camp setting with limited space, loss and theft, and properly dosing the product to last until the next visit. Proposed solutions included storing rations out of children's reach or providing locked boxes in which to keep the supplies, though neither assured complete security.

“Mothers are complaining that when they keep [the rations] at home, they cannot protect them when they are out. The children may come and take the food and misuse it. … This was a complaint.” (NGO Practitioner, Ethiopia, refugee camp)

Similarly, increased sale and intrahousehold ration sharing was commonly reported, though this could be ascribed to increased food insecurity and constrained livelihoods during the pandemic. Increased engagement with caregivers, families and community leaders on the importance of protocol adherence, both at health facilities and at home, was employed by some interviewees to mitigate sales and sharing.

While programme data were not collected for this study, interviewees discussed anecdotal observations on programme performance. Programme staff were consistently concerned about deterioration for clinically vulnerable children, possible increased default rates, and extended LOS. Some interviewees addressed these concerns by conducting phone calls or home visits between follow‐up appointments; however, movement restrictions presented challenges. Another practice was to provide caregivers with additional counselling on monitoring their children (e.g., through Family MUAC and training on danger signs), and consistently emphasise the importance of adhering to dosage schedules and not sharing nutrition supplies.

“I think people are falling sick along the way [in between facility visits] … Previously, they would come and tell you, ‘I am feverish‘, and you would lead them to services immediately. But this time they come at once for the refill and also for treatment, [and regressed] somehow, somewhere, especially where there is malaria and loss of appetite or diarrhea.” (NGO Practitioner, Yemen)

Despite initial concerns, some interviewees reported no observed changes to default rates, nonresponse rates or LOS. One interviewee in South Sudan also proposed that the convenience of reduced follow‐up visits might, with appropriate follow‐up, compensate for other challenges and access barriers. Therefore, increased coverage may ultimately mitigate the potentially negative impacts on caseloads and performance indicators.

## 4 DISCUSSION

This paper presented practitioners' experiences in implementing COVID‐19 mitigation measures in the form of four CMAM protocol adaptations during the pandemic: (1) Family MUAC; (2) modified admission and discharge criteria, including suspended WHZ and/or increased MUAC thresholds; (3) modified dosage of therapeutic foods; and (4) a reduced frequency of follow‐up visits. Our findings demonstrate that practitioners believe the modifications were largely successful in facilitating service continuity and reducing risk of transmitting the virus at health facilities. Staff and caregivers generally accepted well all four programme adaptations during the pandemic.

While the adaptations largely achieved their desired aim, discussions as to which should continue will be context‐specific and must also consider the operational realities of implementation as well as desirable and undesirable unintended consequences (Turcotte‐Tremblay et al., 2021). Operationally, adaptations were widely reported to simplify and streamline programme logistics and implementation, with positive impacts on staff workload and caregiver understanding of the CMAM programme. This could have positive implications for both coverage and resource management, a set of desirable yet, in this context, unintended consequences.

However, several studies have also indicated that COVID‐19 mitigation measures have unintentionally driven significant disruptions to health service provision and uptake (Inzaule et al., 2021; Kotlar et al., 2021; Walker et al., 2020). Within this study, interviewees expressed concern about potential impacts on CMAM programme admissions, children's progress throughout treatment, and programme outcomes; anecdotal validation of these concerns varied across contexts. Finally, the increased home visits and follow‐up that some organisations implemented to mitigate negative impacts of the adaptations may pose additional transmission risks without sufficient PPE and IPC adherence. The lack of programmatic monitoring data compared to a control group highlights the need for more rigorous research and evidence on how these adaptations impact programme outcomes, as well as to document unintended consequences and both optimise protocols and build confidence among implementing staff.

### 4.1 Lessons learned from adaptations

Implementation of Family MUAC during the pandemic demonstrated that it can complement existing screening activities, with the potential to expand screening coverage and fill gaps when routine screenings are insufficient or nonexistent. Furthermore, a benefit of self‐screening approaches is earlier detection, which, when resulting in earlier enrolment in treatment, could result in improved programmatic outcomes (Austoker, 1994; Fleming et al., 2015; Roth et al., 2011). For example, a higher mean MUAC at admission is significant predictor of successful and sustained recovery (Stobaugh et al., 2019), and earlier detection may preclude more severe cases (ALIMA, 2016; Brown et al., 2019; Gnamien et al., 2021). Indeed, previous studies have shown that children referred through Family MUAC were less likely to require inpatient care (Alé et al., 2016).

However, increasing home‐based screening through Family MUAC is one small component of the causal chain between earlier identification and improved treatment outcomes. This study also identified the need for a functioning health system to maximise the benefits of self‐assessment, both in terms of training on self‐screening and referral and service availability. Many caregivers face high opportunity costs to accessing care, a key barrier to health‐seeking behaviours (Ahinkorah et al., 2021; Akinyemi et al., 2019; Blanárová et al., 2016). They may therefore be discouraged from seeking care again if they are turned away after an incorrect measurement or if the services they seek are unavailable. For example, interviewees expressed concern about long‐term caregiver measurement accuracy, contrary to prior studies demonstrating caregiver capacity (Alé et al., 2016; Blackwell et al., 2015; Bliss et al., 2018; Grant et al., 2018). Inaccurate measurements could result from a knowledge decline after initial training, noted in multiple contexts (UNICEF, 2020). The disparities between prior studies and this study could also result from differences between rigorously monitored research and rapidly scaled operational settings. Interviewees emphasised the importance of regular refresher trainings to maintain accurate measurements, as found in other studies (Alé et al., 2016; UNICEF, 2020), requiring strong engagement with health workers and services. Without such engagement, accuracy may decline, resulting in mistaken self‐referrals.

Furthermore, Family MUAC is designed to increase demand for nutrition treatment services but does not necessarily increase service availability and accessibility. Early referral could generate demand for an unavailable service: MAM service coverage is historically low and difficult to quantify (Brown et al., 2019), and there lacks global consensus on optimal MAM treatment (Lelijveld et al., 2019). Any programme that encourages self‐monitoring and self‐referral should therefore also aim to ensure treatment availability for the identified condition.

Interviewees reported that suspending WHZ successfully reduced contact between staff and children. Despite these successes, questions remain regarding optimal criteria to capture vulnerable children and maximise programme coverage, as expressed by interviewees who anticipated returning to the use of WHZ. These debates echo ongoing global‐level conversations about ensuring that programme admissions criteria target children most at risk of mortality or severe illness while practically balancing the demand for supplies to meet caseloads (Aguayo et al., 2015; Briend et al., 2012; Grellety & Golden, 2016, 2018; Rasmussen et al., 2012). Operationally, increasing MUAC thresholds anecdotally increased caseloads and stretched resources, aligning with previous research findings (Guesdon et al., 2021). This illustrates the critical need to identify and optimise inclusion of prioritised groups while accounting for the realities of supply and budget constraints in the context of an abrupt change in targeting criteria, whether for CMAM or other programming.

In general, modifying the dosage of RUTF was a reactive adaptation to compensate for suspended weight measurements. However, it was lauded for its impact on streamlining processes and reducing caregivers' time at facilities, in addition to improving caregivers' understanding of the overall programme. Other studies have suggested that the approach may enable treatment of more children given less food product used per child (Bailey et al., 2020; Daures et al., 2020; Maust et al., 2015; N'Diaye et al., 2021), as commented by one interviewee; however, these data were not collected during this study. While several interviewees expressed concern about potentially negative impacts on children's progress and recovery, multiple studies have shown that recovery rates with varied modified dosages are noninferior to those under standard dosage protocols (Bailey et al., 2020; Kangas et al., 2019; Maust et al., 2015). Further research is needed to understand the full impact of modified dosage on programmatic outcomes and costs, especially at scale.

Finally, reduced frequency of follow‐up visits throughout CMAM treatment enabled social distancing adherence and less crowding at health facilities while also reducing caregivers' need to travel. Supply chain impacts under this model compared to those for standard visits are limited, given that the total amount of product (e.g., RUTF) used per child did not seem to change. Caregivers may also struggle to manage the increased ration distributed at each visit. While the approach largely achieved its mitigation aims, reducing children's contact with the health system without compensatory measures could render them more vulnerable to deterioration, delay concurrent disease identification, and interrupt routine immunisations and supplementation (Inzaule et al., 2021). The evidence base on this adaptation specifically is limited to date (Hanson, 2019; Isanaka et al., 2017). Further programmatic data analysis is necessary to understand if, how, and through what causal link this adaptation could affect health service utilisation and programme outcomes, such as LOS.

### 4.2 Strengths and limitations

A key strength of this study is the breadth of practitioners interviewed, providing insights into the application of these approaches in myriad contexts and offering general takeaways alongside context‐specific findings. However, due to the qualitative and context‐specific nature of these findings, results should be interpreted as indicative only of practitioner experience. Qualitative findings are restricted to those who responded to requests for interview, and therefore, qualitative data was not collected for all adaptations implemented globally. Finally, most interviews took place within a few months of adaptation rollout. Findings therefore strongly reflect experiences in the early stages of implementation and may be less applicable in later stages.

## 5 CONCLUSION

The COVID‐19 pandemic encouraged a paradigm shift in CMAM programming, as pandemic conditions challenged current service provision protocols. This raises the question of which CMAM programme components should be maintained, adapted and strengthened, both during the pandemic and beyond. The protocol adaptations analysed in this study addressed immediate needs for continuity of care while reducing some transmission risks and were mostly well‐accepted by caregivers and staff. However, more rigorous evidence is needed on programme impacts and outcomes.

The pandemic underscored the need for stronger caregiver and community ownership over screening for and referral to acute malnutrition treatment. Movement restrictions and limited transportation led to suspended community screenings and less frequent health worker visits. The responsibility for identifying acute malnutrition therefore shifted largely to caregivers, here illustrated through implementation of Family MUAC. The need to equip caregivers with the necessary tools, information and training to assume this responsibility featured across contexts and programmatic experiences. The approach saw overall acceptance and excitement from healthcare providers and community members and was largely seen as a valuable tool to increase acute malnutrition detection, particularly in contexts with regular gaps in routine screening activities. Further research is needed to identify optimal context‐specific training mechanisms and effective and fair CHW and caregiver incentives, and to understand how the approach can most positively impact programme outcomes, both in the context of COVID‐19 and beyond.

While interviewees reported several positive operational implications of modified admission criteria, modified dosage and reduced frequency of follow‐up visits, there remained widespread concerns about impacts on programme performance, for which rigorous and comparable data is lacking. Top prospective concerns included: excluding vulnerable children by modifying admission criteria; declining programme outcomes associated with a modified dosage; and deterioration and poorer outcomes associated with a reduced frequency of visits. Still, these concerns were not quantitatively validated during this study. Nonetheless, most interviewees reported that, despite the strengths of some adaptations, they anticipated reverting to standard protocols once COVID‐19 transmission risks were mitigated. Multi‐year programmatic data analysis and rigorous research in diverse contexts is critical to confirm or contradict these concerns and inform decision‐making.

Beyond acute malnutrition screening and treatment, these findings have illuminated several larger implications for adaptive programming in the face of disruption. First, it is critical to be mindful of and proactively address possible negative impacts or unintended consequences that may arise from adaptations. For example, relying solely on low‐literate caregivers for community‐level screening may broaden the gap between those who do and do not receive treatment, as more educated caregivers may be better positioned to rigorously monitor MUAC. Reducing the frequency of follow‐up visits may systematically disadvantage children who already have limited access to health and nutrition services, placing vulnerable children at greater risk of illness and death. Responsive and adaptive programming must prioritise equity and inclusion, despite facing multiple challenges.

Finally, the pandemic demonstrated once again that necessity spurs innovation: these approaches had been piloted previously but were implemented at a greater scale than ever before due to COVID‐19 restrictions. During the pandemic, donors and governments created an enabling environment in which to challenge the status quo, with prospectively positive implications for programming beyond COVID‐19. Moving forward, such creative thinking should not be exclusively responsive. Rather, donors and organisations should continue to encourage flexibility and experimentation, strengthening the evidence base on existing adaptations while laying the foundation for new and unexpected ideas from implementing organisations and communities alike. As demonstrated by the discussions and rollout during the pandemic, such adaptability requires close collaboration and communication between stakeholders at all levels—communities, national implementing partners and coordination mechanisms, and global agencies. Strengthening these relationships and lines of communication during calmer times can enable preparation for the unexpected.

## AUTHOR CONTRIBUTIONS

Heather Stobaugh and Sarah King conceptualized and designed the study and sought ethical approval. Heather Stobaugh, Sarah King and Ronald Stokes‐Walters developed the study surveys and interview guides. Maria Wrabel, Ronald Stokes‐Walters, Heather Stobaugh and Sarah King conducted the interviews. Maria Wrabel and Ronald Stokes‐Walters developed the analysis plan and led analysis. Maria Wrabel and Ronald Stokes‐Walters led the writing of the manuscript. Sarah King and Heather Stobaugh provided revision and critical feedback on all drafts of the paper. Grace Funnell provided input into the study design and feedback into the final manuscript.

## CONFLICT OF INTEREST

The authors declare no conflict of interest.

## ETHICS STATEMENT

The study protocol was approved by Solutions IRB (Reference #2020/06/18). All participants gave written informed consent before agreeing to take the initial screening surveys. Those individuals contacted for interviews then gave additional verbal consent before beginning their interviews. The funders had no role in the design and execution of the study.

## Data Availability

The data that support the findings of this study are available from the corresponding author upon reasonable request. The data are not publicly available due to privacy or ethical restrictions.
